# Pulmonary Tuberculosis Notification Rate Within Shenzhen, China, 2010-2019: Spatial-Temporal Analysis

**DOI:** 10.2196/57209

**Published:** 2024-06-14

**Authors:** Peixuan Lai, Weicong Cai, Lin Qu, Chuangyue Hong, Kaihao Lin, Weiguo Tan, Zhiguang Zhao

**Affiliations:** 1 Shenzhen Center for Chronic Disease Control Shenzhen China; 2 School of Public Health Sun Yat-sen University Guangzhou China

**Keywords:** tuberculosis, spatial analysis, spatial-temporal cluster, Shenzhen, China

## Abstract

**Background:**

Pulmonary tuberculosis (PTB) is a chronic communicable disease of major public health and social concern. Although spatial-temporal analysis has been widely used to describe distribution characteristics and transmission patterns, few studies have revealed the changes in the small-scale clustering of PTB at the street level.

**Objective:**

The aim of this study was to analyze the temporal and spatial distribution characteristics and clusters of PTB at the street level in the Shenzhen municipality of China to provide a reference for PTB prevention and control.

**Methods:**

Data of reported PTB cases in Shenzhen from January 2010 to December 2019 were extracted from the China Information System for Disease Control and Prevention to describe the epidemiological characteristics. Time-series, spatial-autocorrelation, and spatial-temporal scanning analyses were performed to identify the spatial and temporal patterns and high-risk areas at the street level.

**Results:**

A total of 58,122 PTB cases from 2010 to 2019 were notified in Shenzhen. The annual notification rate of PTB decreased significantly from 64.97 per 100,000 population in 2010 to 43.43 per 100,000 population in 2019. PTB cases exhibited seasonal variations with peaks in late spring and summer each year. The PTB notification rate was nonrandomly distributed and spatially clustered with a Moran *I* value of 0.134 (*P*=.02). One most-likely cluster and 10 secondary clusters were detected, and the most-likely clustering area was centered at Nanshan Street of Nanshan District covering 6 streets, with the clustering time spanning from January 2010 to November 2012.

**Conclusions:**

This study identified seasonal patterns and spatial-temporal clusters of PTB cases at the street level in the Shenzhen municipality of China. Resources should be prioritized to the identified high-risk areas for PTB prevention and control.

## Introduction

Tuberculosis (TB), caused by the bacillus *Mycobacterium tuberculosis*, is a chronic communicable disease that is a major public health and social problem and one of the leading causes of death from a single bacterial pathogen worldwide [1]. According to the World Health Organization (WHO) 2023 report, there were 7.5 million new TB cases and 1.3 million deaths caused by TB estimated in 2022 globally [1]. The 30 high-TB-burden countries accounted for 87% of all estimated incident cases worldwide [1].

China had the third highest TB burden in the world in 2022, with an estimated 530,000 new cases of TB, accounting for 7.1% of the global total [1]. To reduce the magnitude and burden of TB, China has implemented several effective interventions such as the adoption and implementation of the End-TB Strategy, expansion of directly observed therapy strategies (DOTS), and expansion of multidrug-resistant TB diagnosis and treatment centers. As a result, China has made great progress in reducing the annual TB incidence by 3.4% since 1990 [2]. However, TB remains a major public health problem in China.

Over the past few decades, China’s urbanization process, led by nationwide economic reforms, has been accompanied by the rapid growth of cities, including Shenzhen, due to the migration of the younger population from rural areas owing to more attractive employment opportunities [3], contributing to large-scale domestic migrations. Rural labor migrants might change TB transmission patterns in the cities; moreover, the health problems of migrants, including TB infection and active disease development, might change under the new circumstance of living in the city [4]. As a special economic zone, Shenzhen has experienced remarkable development in terms of both the economic scale and population growth. However, the massive influx of migrants to Shenzhen has brought serious challenges for the prevention and control of TB [3].

Identifying areas where TB is geographically concentrated is particularly essential for the planning, implementation, monitoring, and evaluation of TB control programs and to inform the allocation of resources for targeted and effective interventions [5]. In recent years, spatial-temporal analysis has been widely used to describe the distribution characteristics and transmission patterns of TB, demonstrating that TB exhibits highly dynamic and spatially heterogeneous patterns at provincial, national, and international levels during certain periods of time [6,7]. However, few studies have been performed to reveal the changes in the small-scale clustering of TB cases at the street level. In this study, we performed a spatial-temporal cluster analysis to characterize the distribution and patterns of pulmonary tuberculosis (PTB) cases at the street level in the Shenzhen municipality of China from 2010 to 2019.

## Methods

### Study Setting

Shenzhen is a city located in southern China at a longitude of 113°43′-114°38′ East and a latitude of 22°24′-22°52′ North (see Multimedia Appendix 1), with 74 streets among its 10 districts. Shenzhen has greatly benefited from the implementation of reforms and opening-up policies of China in the 1980s and developed quickly from a small fishery village of less than 1 million inhabitants to a modern metropolis with over 10 million permanent residents, making it one of the most developed municipalities in China [8]. Notably, over 70% of the residents in Shenzhen are migrants and most of these migrants are young, with those 65 years and older accounting for only 3.22% of the population [8]. The gross domestic product (GDP) of Shenzhen has increased by more than 110,000 times in the past 40 years, from less than 200 million yuan in 1979 (~US $28 million) to approximately 2.69 trillion yuan in 2019 (~US $370 billion) [8].

### Data Source

Data of reported PTB cases in Shenzhen from January 2010 to December 2019 were extracted from the China Information System for Disease Control and Prevention, which is established and operated by the Chinese Center for Disease Control and Prevention (CCDC). Each reported case contains demographic and medical information and is identified by a unique identity card number to avoid duplicate reports.

The annual population data of each administrative street for each year were extracted from Shenzhen Statistical Yearbooks (2010-2019) of the Shenzhen Municipality Bureau of Statistics [8]. Basic map data were obtained from the Shenzhen Geographical Information Public Service Platform. The administrative number of the street was used as a reference at a 1:500,000 scale to correlate and match the data of reported PTB cases on each street to establish the database.

As one of the most serious infectious diseases in China, each diagnosed PTB case must be reported online within 24 hours after diagnosis. The Diagnostic Criteria for PTB (WS288-2017) issued by the Ministry of Health (the former National Health Commission) of the People’s Republic of China in 2017 were used in this study, in which cases of PTB were diagnosed using radiography, pathogen detection, and pathologic diagnosis [9]. All forms of PTB were included in this study, including bacteriologically confirmed and clinically diagnosed PTB, previously treated and new PTB, and childhood and adult PTB.

### Ethical Considerations

This study was approved by the Ethics Committee of Shenzhen Center for Chronic Disease Control (SZCCC-2023-003-01-PJ). Personal sensitive information such as the name and phone number associated with each case were blocked and excluded in this study to protect the patients’ privacy. A secondary analysis based on reported data was conducted and thus informed consent from individuals was not required. This study was conducted strictly in accordance with Norms for the Management of Information Reporting on Infectious Diseases in China [10].

### Data Analysis

#### Descriptive and Time-Series Analyses

The epidemiological characteristics of PTB cases were analyzed. PTB cases reported from 2010 to 2019 were aggregated annually by age, sex, occupation, permanent residents and migrant population, and date of onset for PTB notification rate analysis. Comparison between different groups was performed using the χ^2^ test or Fisher exact test as appropriate. All statistical analyses were performed in SPSS version 26 and a two-tailed *P* value less than .05 was considered statistically significant.

In addition, time-series seasonal decomposition analysis was used to identify the seasonality of the PTB notification rate in Shenzhen municipality. The time series of reported PTB cases were decomposed into seasonal variation, long-term trend, and random effect to explore the temporal patterns. The temporal patterns were determined according to the onset date of the monthly registration of all reported PTB cases. The time series included 120 months from January 2010 to December 2019 and were examined using Excel software.

#### Spatial Autocorrelation Analysis

Moran *I* was selected as the global index of spatial autocorrelation to detect the spatial distribution pattern of PTB cases in Shenzhen municipality, China. The expression of the global Moran *I* statistic is as follows:

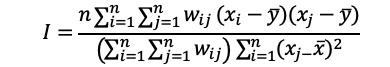



where *n* is the number of units and *x_i_* and *x_j_* are the attribute values of unit *i* and unit *j*, respectively. The Moran *I* value varies between –1 and 1; when |*I*| is larger, the autocorrelation is higher, and *I*=0 indicates no autocorrelation [11]. Both the *Z* score and *P* value calculated by GeoDa 1.20 (Arizona State University) were used to test the significance of the Global Moran *I* value.

#### Spatial-Temporal Scan Analysis

Kulldorff spatiotemporal scan statistical analysis was performed to explore the spatial, temporal, and spatial-temporal clusters of PTB across different streets geographically and at different time periods [12]. The SaTScan 10.1 software (Kulldorff) was used for spatial-temporal scanning analysis, which is based on a moving-column scanning window that contains geographical information with the height corresponding to time [13]. According to a previous study [6], the maximum radius of the spatial-scanning window and the maximum length of the temporal-scanning window was set to 11% of the population at risk and 30% of the whole study period, respectively. The log likelihood ratio (LLR) of different circle centers and various radii was calculated as follows to compare the notification rate of PTB inside and outside the scanning window [14]:

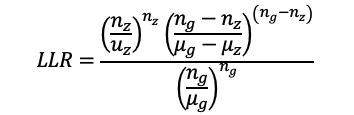



Where *n_z_* signifies the observed count of events within spatiotemporal window *z*, *n_g_* denotes the total aggregate of events across the entirety of the study area, *u_z_* represents the expected count of events within the particular spatiotemporal window *z*, and *μ_g_* indicates the overall anticipated count of events within the study area. A larger LLR value indicates a more likely cluster. The Monte Carlo simulation test was used to evaluate whether the likelihood of the cluster was statistically significant, and the relative risk (RR) was calculated as the estimated risk within the cluster divided by that outside the cluster [15,16]. For each possible spatiotemporal cluster, when the *P* value is less than .05, a larger LLR value indicates that the area covered by the dynamic scanning window is more likely to be a cluster region. The window with the largest LLR value selected from all the scanning windows is considered the strongest cluster and the secondary clusters are the other windows with statistically significant LLR values. ArcMap software (Esri) was used to visualize the scanning results.

## Results

### Descriptive Analysis of PTB Cases

**Figure 1:**
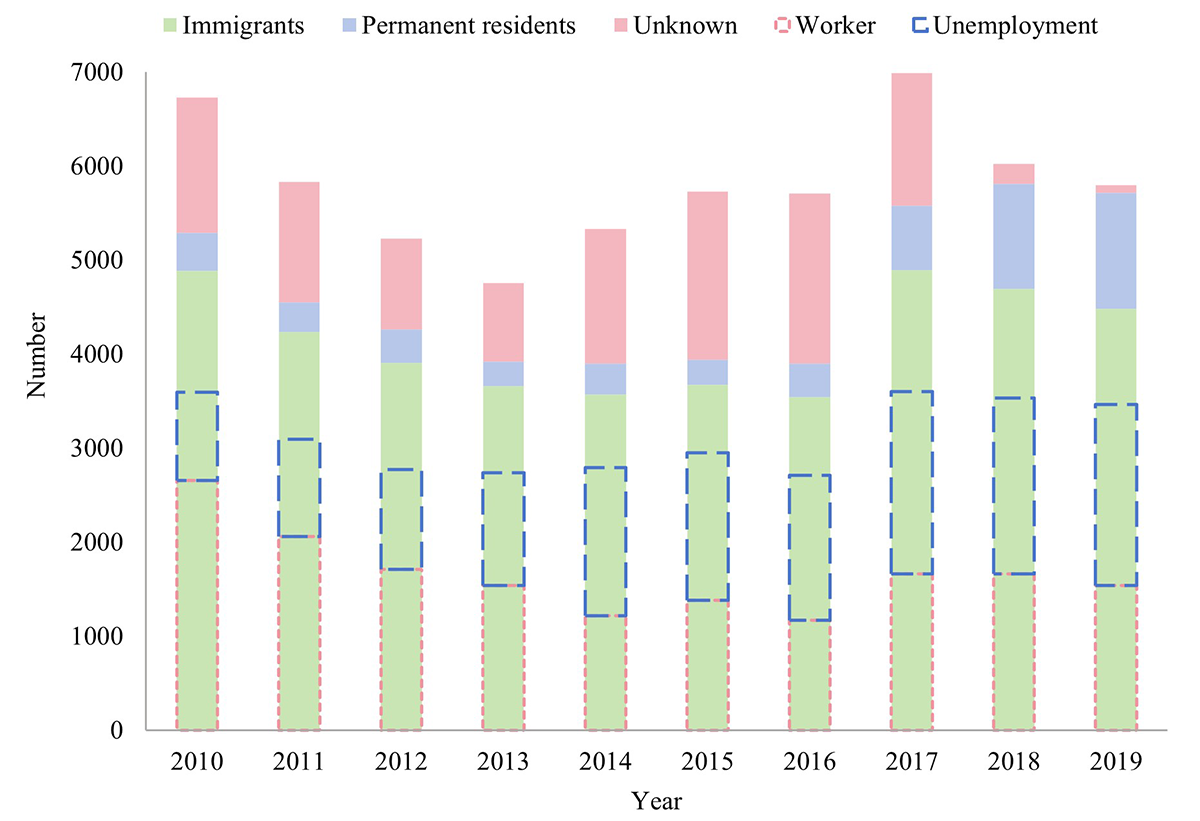
Characteristics of pulmonary tuberculosis cases from 2010 to 2019 in Shenzhen municipality, China.

A total of 58,122 PTB cases were notified in Shenzhen from 2010 to 2019, including 38,358 (66.00%) cases from male patients. Over 60% of cases were from the migrant population; 20,795 cases (35.78%) were detected in workers and 20,629 (35.49%) were from individuals who were unemployed. The mean age of the patients diagnosed with PTB was 34.73 (SD 14.13) years, with a range from 0 to 97 years (Figure 1).

### Temporal Patterns of PTB Cases

**Figure 2:**
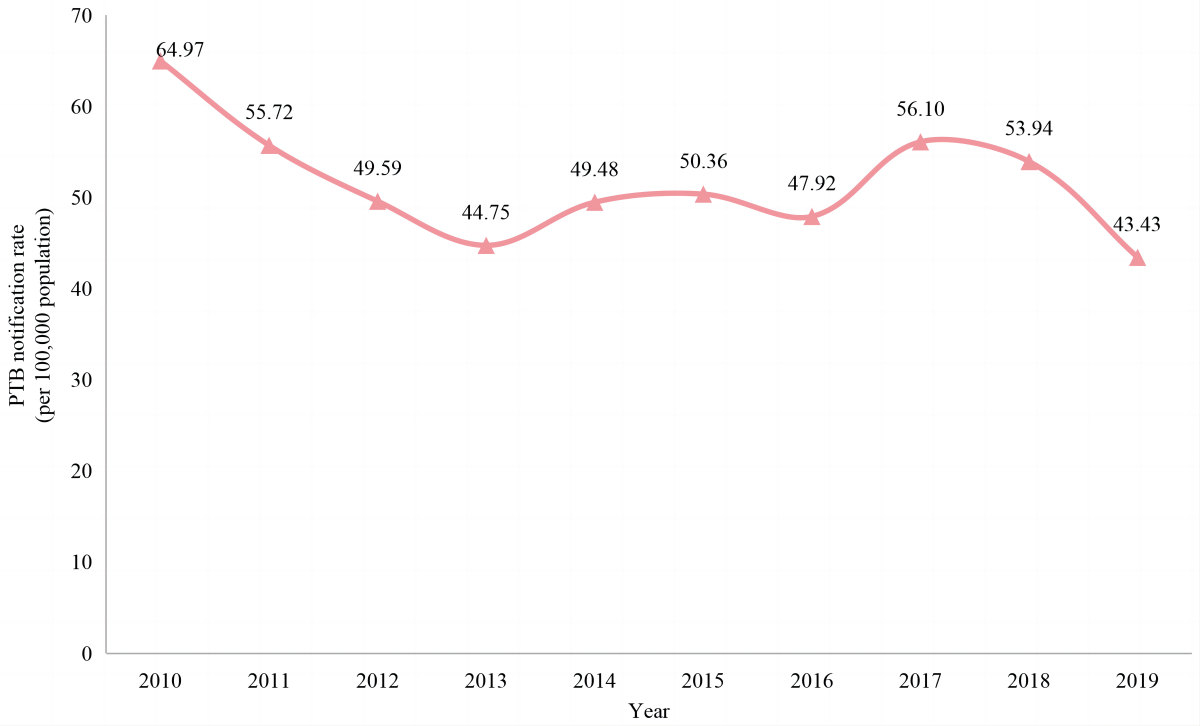
The variation trend of pulmonary tuberculosis (PTB) notification rate (per 100,000 population) from 2010 to 2019 in Shenzhen municipality, China.

The annual average notification rate of PTB for the 10-year study period was 51.62 per 100,000 population. As shown in Figure 2, the total notification rate of PTB showed a significantly volatile downward trend over time from the highest rate of 64.97 per 100,000 population in 2010 to the lowest rate of 43.43 per 100,000 population in 2019 (*P*<.001).

**Figure 3:**
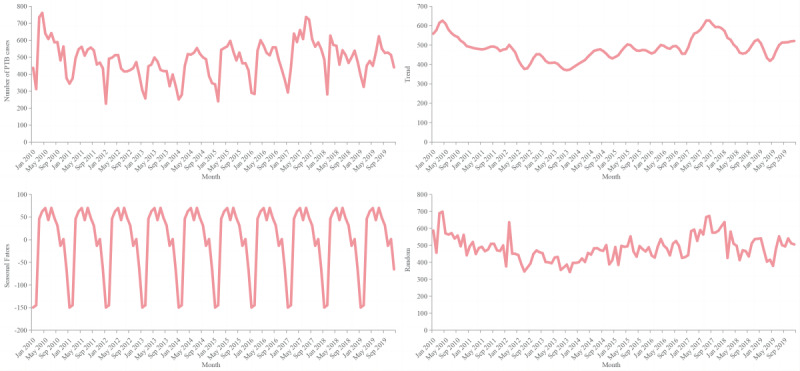
The seasonal distribution of monthly pulmonary tuberculosis (PTB) cases from 2010 to 2019 in Shenzhen municipality, China.

The monthly notification rates of PTB showed a trend of volatility and decline over the study period in Shenzhen municipality. PTB cases have shown seasonal variations with the highest number of PTB cases notified in May and July each year, followed by a steady decreasing trend after July and a nadir in January and February (Figure 3).

### Spatial Patterns of PTB Cases

As displayed in Table 1, the global spatial autocorrelation analysis showed that the Moran *I* value of the PTB notification rate in Shenzhen municipality from 2010 to 2019 was positive (0.134) and the *P* value was consistently less than .05, indicating that the notification rate of PTB in Shenzhen municipality was nonrandomly distributed. Annually, except for the years 2012 and 2014, there was significant global spatial autocorrelation in the PTB notification rates every year with the Moran *I* value ranging from 0.130 to 0.353. Therefore, further spatial-temporal scan analysis of PTB was needed.

**Table 1:** Global spatial autocorrelation analysis of the pulmonary tuberculosis notification rate from 2010 to 2019 in Shenzhen municipality, China.

Year	Moran *I* value	*Z* score	*P* value^a^
2010	0.353	5.320	.001
2011	0.179	2.748	.002
2012	0.095	1.398	.07
2013	0.143	2.103	.02
2014	0.047	0.894	.19
2015	0.130	1.851	.03
2016	0.190	2.786	.006
2017	0.217	3.065	.002
2018	0.201	2.852	.002
2019	0.155	2.361	.02
2010-2019	0.134	1.894	.02

^a^The autocorrelation is considered significant at *P*<.05 (two-tailed).

### Spatial Clustering of PTB Cases

**Figure 4:**
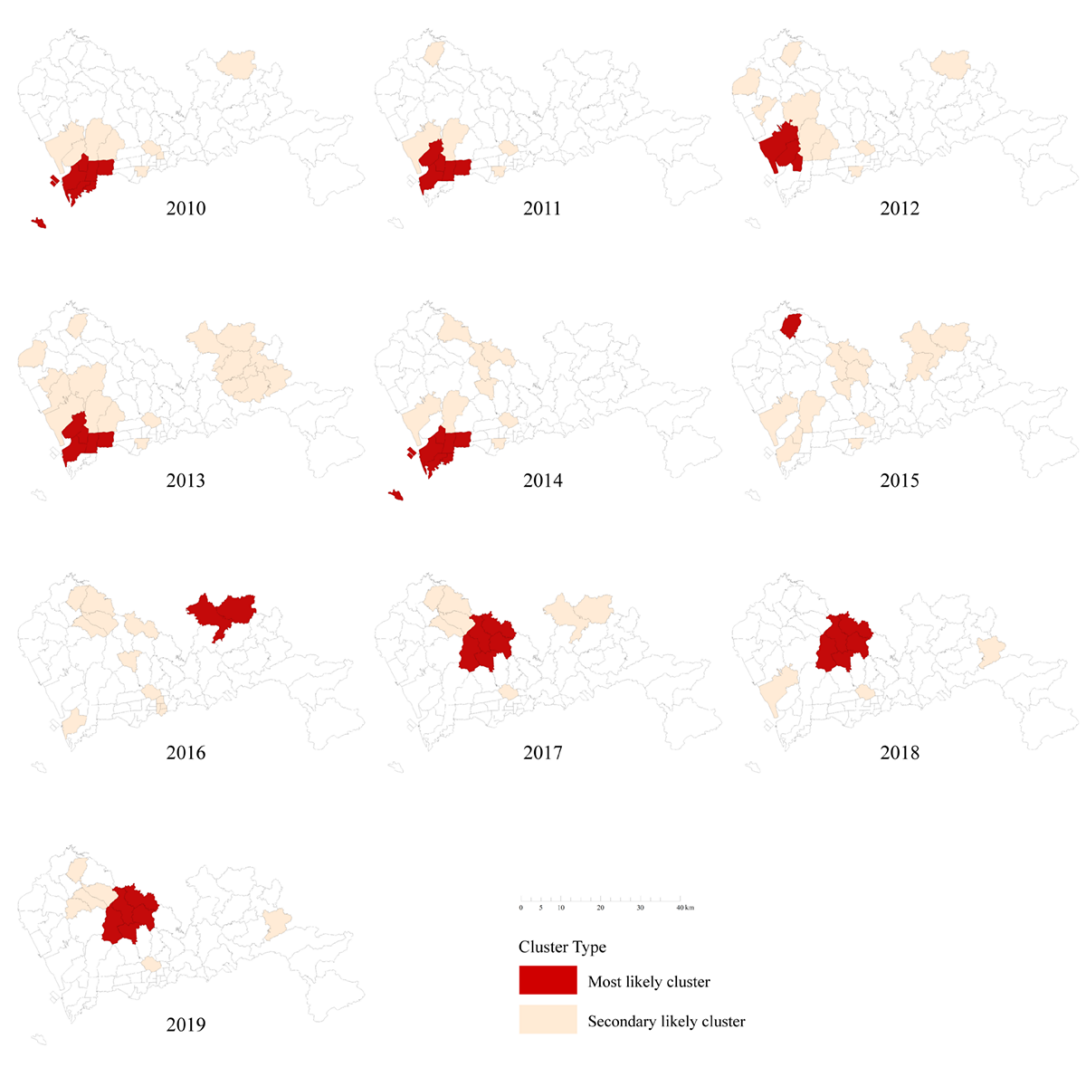
The spatial clusters of notified pulmonary tuberculosis (PTB) cases from 2010 to 2019 in Shenzhen municipality, China.

Spatial clustering analysis of notified PTB cases every year from 2010 to 2019 showed that the most likely clusters have changed dynamically over time. The most likely clusters of PTB in Shenzhen municipality from 2010 to 2014 were concentrated in the southwest regions, covering the streets of Nanshan District and Baoan District. From 2015 to 2019, the most likely clusters were mainly distributed in the central and northern streets of Shenzhen municipality, including Longhua District, Longgang District, and Guangming District (Figure 4).

### Temporal Clustering of PTB Cases

As presented in Table 2, the temporal clustering analysis in each year showed that PTB notification rates were concentrated in late spring and summer annually, ranging from March to August. The greatest aggregated time for PTB across the whole study period was observed from March 2010 to September 2010. During this period, there was a total of 4562 notified PTB cases and the risk of PTB-related incidents was 53% (RR 1.53, *P*<.001) higher than that in other time periods.

**Table 2:** Temporal clustering of notified pulmonary tuberculosis cases from 2010 to 2019 in Shenzhen municipality, China.

Year	Cluster time frame	Observed cases	Expected cases	RR^a^	LLR^b^	*P* value^c^
2010	March 1 to May 31	2136	1696.58	1.38	72.30	<.001
2011	July 1 to September 30	1642	1469.98	1.16	13.13	<.001
2012	February 1 to April 30	1500	1286.07	1.23	22.79	<.001
2013	April 1 to June 30	1430	1185.74	1.29	32.14	<.001
2014	June 1 to August 31	1600	1344.21	1.27	31.31	<.001
2015	April 1 to June 30	1714	1428.82	1.28	36.40	<.001
2016	April 1 to June 30	1693	1418.70	1.27	33.92	<.001
2017	July 1 to August 31	1458	1186.66	1.29	35.34	<.001
2018	March 1 to May 31	1766	1517.62	1.23	26.28	<.001
2019	June 1 to August 31	1698	1460.91	1.23	24.88	<.001
2010-2019	March 1 to September 30, 2010	4562	3072.58	1.53	334.01	<.001

^a^RR: relative risk.

^b^LLR: log likelihood ratio.

^c^Considered significant at *P*<.05 (two-tailed).

### Spatial-Temporal Clustering of PTB Cases

**Figure 5:**
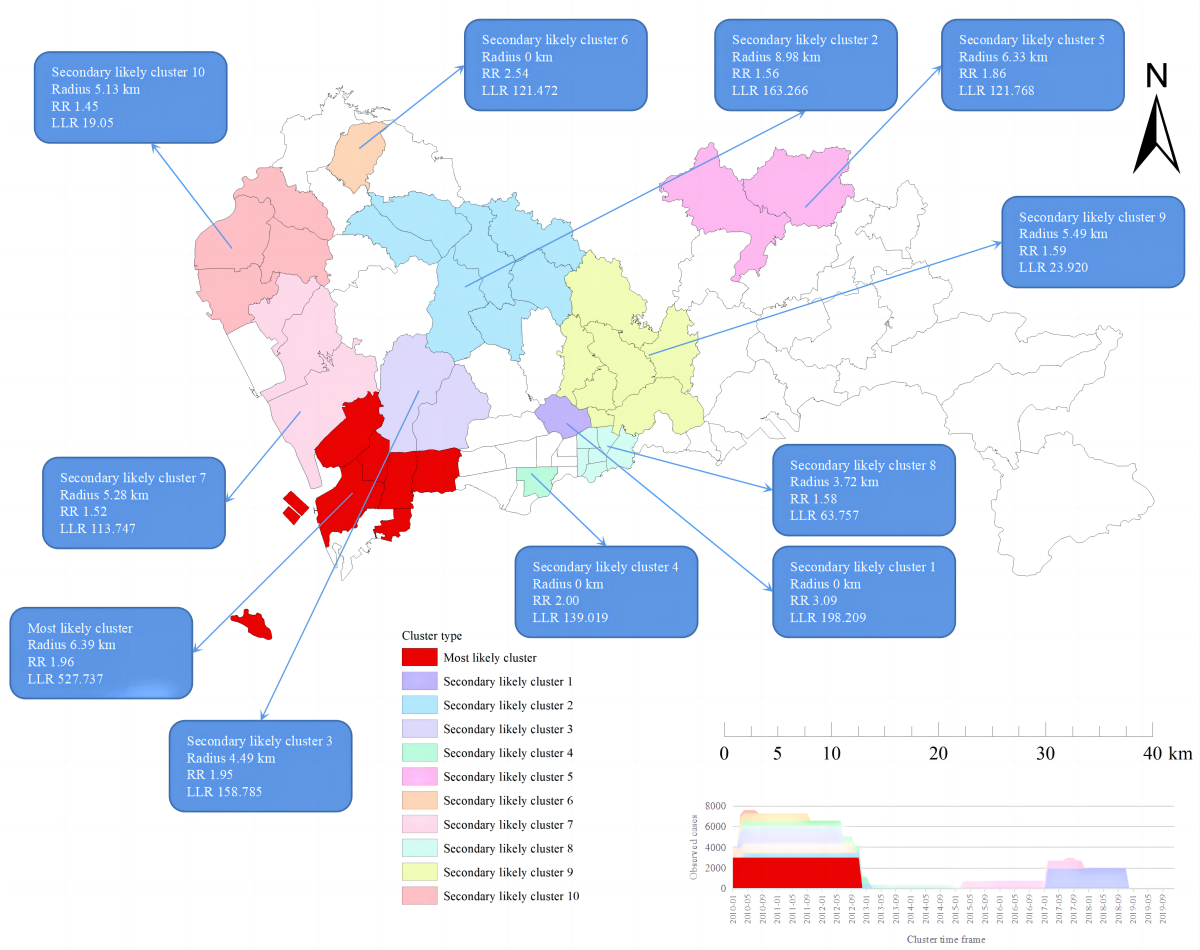
Spatial-temporal clustering of notified pulmonary tuberculosis (PTB) cases from 2010 to 2019 in Shenzhen municipality, China. LLR: log likelihood ratio; RR: relative risk.

The results of spatial-temporal clustering analysis for notified PTB cases in Shenzhen municipality from 2010 to 2019 are shown in Table 3 and Figure 5, indicating that the PTB notification rates were spatiotemporally clustered. A total of 1 most-likely cluster and 10 secondary clusters were detected in this study. The most-likely cluster area was distributed in the southwestern region of Shenzhen municipality and the clustering time was from January 2010 to November 2012 (RR 1.96, *P*<.001) with a total of 3000 PTB cases notified during this period. In addition, the area centered at Nanshan Street of Nanshan District (22.53 N, 113.94 E) with a radius of 6.39 kilometers covered 6 streets, similar to the most likely cluster identified in the purely spatial clustering analysis. The other 10 secondary clusters were mainly located in the central and northwestern regions of Shenzhen municipality, covering streets in Baoan District, Nanshan District, Longgang District, Guangming District, and Longhua District, along with several relatively small areas (clusters 1, 4, and 8) in Futian District and Luohu District. The main clustering time ranged from January 2010 to March 2013 except for the secondary clusters 2, 5, 6, and 9.

**Table 3:** Spatial-temporal clustering of notified pulmonary tuberculosis cases from 2010 to 2019 in Shenzhen municipality, China.

Cluster type	Cluster time frame	Coordinates/radius (km)	Prefectures, n	Observed cases	Expected cases	RR^a^	LLR^b^	*P* value^c^
Most likely cluster	January 1, 2010, to November 30, 2012	22.53 N, 113.94 E/6.39	6	3000	1573.33	1.96	527.74	<.001
Secondary cluster 1	April 1, 2010, to February 28, 2013	22.58 N, 114.09 E/0	1	441	143.58	3.09	198.21	<.001
Secondary cluster 2	February 1, 2017, to November 30, 2018	22.72 N, 114.02 E/8.98	7	1943	1258.93	1.56	163.27	<.001
Secondary cluster 3	January 1, 2010, to September 30, 2012	22.58 N, 113.99 E/4.49	2	888	458.81	1.95	158.79	<.001
Secondary cluster 4	March 1, 2010, to January 31, 2013	22.52 N, 114.07 E/0	1	728	366.75	2.00	139.02	<.001
Secondary cluster 5	March 1, 2015, to November 30, 2017	22.77 N, 114.31 E/6.33	2	772	416.77	1.86	121.77	<.001
Secondary cluster 6	March 1, 2013, to November 30, 2015	22.78 N, 113.90 E/0	1	373	147.19	2.54	121.47	<.001
Secondary cluster 7	March 1, 2010, to June 30, 2012	22.63 N, 113.84 E/5.28	3	1516	1006.46	1.52	113.75	<.001
Secondary cluster 8	March 1, 2010, to September 30, 2011	22.55 N, 114.14 E/3.72	6	706	447.87	1.58	63.76	<.001
Secondary cluster 9	July 1, 2017, to September 30, 2017	22.63 N, 114.14 E/5.94	7	261	164.74	1.59	23.92	<.001
Secondary cluster 10	March 1 to July 31, 2010	22.73 N, 113.81 E/5.13	4	317	219.60	1.45	19.05	<.001

^a^RR: relative risk.

^b^LLR: log likelihood ratio.

^c^Statistical significance is indicated at *P*<.05 (two-tailed).

## Discussion

### Principal Findings

To the best of our knowledge, this is the first study to present a spatial-temporal cluster analysis of PTB at the street level in Shenzhen municipality using routinely collected PTB surveillance data, which identified seasonal patterns and spatial-temporal clusters of PTB in Shenzhen.

The notification rate of PTB in Shenzhen municipality declined steadily during the 10-year study period, decreasing from the peak of 64.97 cases per 100,000 population in 2010 to the lowest of 43.43 cases per 100,000 population in 2019. This downward trend is in line with other municipal, provincial, and national studies [6,17-20]. This achievement is due in large part to the great importance attached by the local government and health administrative department in Shenzhen to PTB control and prevention in recent years. First, financial support received from the government has increased continuously from 28.2 million yuan (~US $3.9 million) in the Twelfth Five-Year Plan (2011-2015) to approximately 197.64 million yuan (~US $27 million) in the Thirteenth Five-Year Plan (2016-2020) for TB control and prevention in Shenzhen. Subsequently, an integrated system under collaboration of the CCDC, tuberculosis-designated hospitals, and community health service centers was established and developed among institutions with a clear division of labor and coordination [21]. In addition, a batch of molecular biological testing equipment for PTB diagnosis and treatment was purchased and distributed to designated PTB medical institutions in various streets and districts of the city [21]. As a result, the diagnosis time of PTB and the possibility of pretreatment transmission have declined, despite an increase in the overall percentage of cases detected and confirmed bacteriologically. At the same time, PTB screening programs were added to the routine physical examination for the older population aged ≥65 years and for primary and secondary school students, and rifampicin resistance testing was further strengthened for patients with PTB [22]. In addition to the DOTS recommended by the WHO since 2000, Shenzhen has implemented a series of measures, including providing free drug treatment and a certain degree of reimbursement for examination costs, which enable early diagnosis and timely treatment for patients with PTB to effectively curb the spread of disease. With the implementation of these targeted and effective measures, the PTB epidemic situation has improved substantially, although it remains a great public health challenge in Shenzhen municipality.

According to the time-series analysis, seasonal variations and cyclical trends of PTB cases were observed, with apparent peaks in late spring and summer, especially in May and July. This is consistent with the findings of neighboring Guangzhou city [23]. Shenzhen has a subtropical monsoon humid climate, a long summer and short winter, abundant sunshine, and abundant rainfall, with an average annual temperature of 20 °C and relative humidity of 80%. This is aligned with the transmission of respiratory infectious diseases and may be related to the spread of PTB. Previous studies indicated that lack of exposure to ultraviolet rays from sunlight and poor ventilation in the indoor environment may increase the risk of PTB infection [24-26]. Consistent with other reports [18,19], we found the lowest number of notified PTB cases in January and February. On the one hand, this may be closely related to the massive public transportation and population flow during the Chinese Spring Festival, which usually occurs in late January or February [23,27], when many migrants leave Shenzhen to return to their hometowns. On the other hand, people are busy celebrating the Lunar New Year at this time, coupled with frequent visits to relatives and friends, and thereby may avoid seeking medical care [18,19]. These multiple factors would result in a significant decline in PTB cases reported but an increased risk of transmission among the infectious source and close contacts during the holiday, concentrating in late spring and summer after an incubation period of several months to half a year, including a 2-month interval from symptom appearance to a medical diagnosis [25]. This may be one of the contributors for the peak months observed in Shenzhen. In addition, the return of migrants and delayed diagnosis should also be considered. Therefore, the detection and tracking of patients with PTB should be strengthened during the Spring Festival, and suspected or confirmed cases should be transferred to designated medical institutions for further diagnosis and treatment as soon as possible to shorten the delay time of treatment and reduce the risk of transmission. Health education should also be emphasized and effective measures such as actively wearing masks and opening windows for ventilation should be promoted, especially in crowded places.

Many studies have confirmed a spatial-clustering distribution of PTB in certain areas [6,18,19,28]. In this study, the global spatial autocorrelation analysis indicated that the notification rate of PTB in Shenzhen municipality displayed an obvious spatial-clustering distribution between 2010 and 2019. The results of advanced local spatial autocorrelation showed that the clusters of PTB at the street level had a dynamic change over time from the southwest to the central and northern part of the city. The hot spots of PTB observed in this study are largely consistent with the PTB notification rates in Shenzhen. PTB has long been known as “the disease of the poor,” and poverty has been considered one of the causes of disease clustering [28-31]. However, a previous study indicated that the incidence of PTB is higher in areas with a better economic situation than in less developed areas because the former areas are more attractive to population inflows [32]. Many migrants are at increased risk of ill health because of the adverse conditions through which they travel and then work and live [33]. Migrant workers are usually not entitled to the social welfare and health resources that are available to local permanent residents, which poses a challenge for this specific population in accessing health care services [34,35]. In this study, most of the notified PTB cases were detected in workers or unemployed individuals, shifting in parallel with the economic development of local areas of the city. The regional economic development in the past 10 years started from Nanshan District and Baoan District in southwest Shenzhen, where the GDP ranked in the top three during the study period. With the change of urban planning, the GDP in central and northern Shenzhen, including Longhua District, Longgang District, and Guangming District, has grown rapidly recently, which has brought about a more obvious population agglomeration effect in these areas [36]. Similar dynamics can also be found in Beijing [37] and Shanghai [38]. Understanding the interaction of TB transmission, population migration, and social development, albeit highly complex and dynamic, is essential for the control and prevention of TB [32].

To consider the role of time when evaluating the geographical distribution of PTB, spatial-temporal scanning analysis was used to supplement the local spatial analysis. The results from the spatial-temporal clustering analysis of the PTB cases from 2010 to 2019 showed that the most likely cluster was concentrated in southwestern Shenzhen, covering 6 streets of 2 districts, and the clustering period was from January 2010 to November 2012. In addition, most of the secondary clustering areas were also found in this period, indicating that this period represents a peak of PTB transmission in Shenzhen. The clusters identified by the spatial-temporal scanning analysis and the simple spatial clustering analysis were similar, which may indicate the robustness of our results. More importantly, these similar findings indicate that more effective and targeted measures should be urgently developed and implemented in the high-risk areas for PTB control and prevention in Shenzhen municipality.

Compared with the Guangdong provincial and China national findings in recent years [39,40], Shenzhen has entered a period with a low epidemic level of PTB. However, there is still a long way to go to reach the WHO’s goal of eliminating PTB. Achieving this ambitious goal requires stronger, more tailored and effective responses. First, the clustering results can serve as a guide to develop more accurate and effective interventions, with a focus on areas where PTB is concentrated, and to strengthen the deployment and implementation of corresponding actions to prevent and discover the outbreak and epidemic status of PTB at the street level. PTB diagnosis, treatment, and care for migrants should be integrated into the general health services, while special efforts may be needed to reach migrants for improving the availability, accessibility, and quality of comprehensive medical services [41]. Screening for PTB contacts and selected high-risk groups should be linked to follow-up, strategies for preventive treatment, or referral to the treatment program [41]. In addition, systematic and extensive health educational campaigns are needed for the provision of more accessible information to raise public awareness of PTB and improve public access to relevant health care services. Third, increasing the speed of referral of newly detected patients with PTB to designated clinics is also crucial to minimize the risk of disease transmission and infections in the community.

### Limitations

This is the first study to analyze the spatial-temporal clustering characteristics of PTB at the street level and identify the high-risk areas of PTB in Shenzhen municipality, which provides valuable information for future strategies and measures of PTB prevention and control. However, this study was subject to several limitations. First, our analysis was based on data extracted from the National Surveillance System and we were unable to preclude the possibility of missing cases. This might cause an underestimation of the PTB epidemic in Shenzhen. Second, this study only focused on the spatial and temporal patterns and clusters of PTB cases. Potential risk factors associated with PTB incidence, such as individual habits, socioeconomic status, living conditions, and environmental pollutants, were not evaluated. Further research should take these limitations into consideration.

### Conclusions

This study identified spatial and temporal patterns and spatial-temporal clusters of PTB cases at the street level in Shenzhen municipality from 2010 to 2019. A volatile downward trend of PTB incidence over the study period was observed in Shenzhen municipality. The most likely clustering areas changed from the southwest to the central and northern part of the city, and the most likely clustering time was late spring and summer. Resources should be prioritized to high-risk areas for PTB prevention and control.

## Appendix

Multimedia Appendix 1 Location of Shenzhen municipality in China.

## Abbreviations

CCDC: Chinese Center for Disease Control and Prevention
DOTS: directly observed therapy strategy
GDP: gross domestic product
LLR: log likelihood ratio
PTB: pulmonary tuberculosis
RR: relative risk
TB: tuberculosis
WHO: World Health Organization

## Data Availability

The data sets used and/or analyzed during the current study are available from the corresponding author on reasonable request.
