# Supplementary figures and images for: Pulmonary Tuberculosis Notification Rate Within Shenzhen, China, 2010-2019: Spatial-Temporal Analysis
Source: JMIR Public Health Surveill. 2024 Jun 14;10:e57209. doi: 10.2196/57209 (PMC11214025; doi:10.2196/57209)

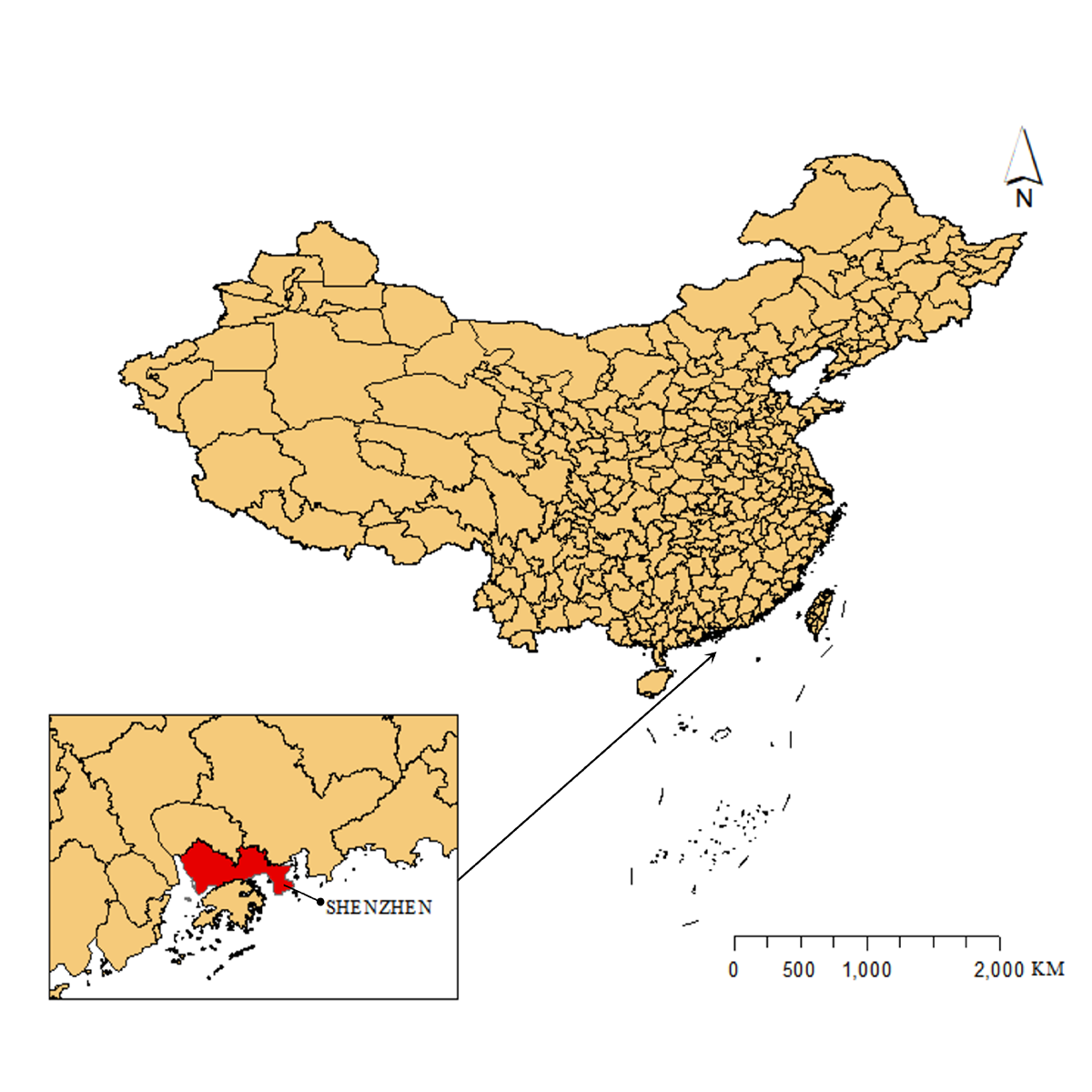

Supplement: Multimedia Appendix 1 [file publichealth_v10i1e57209_app1.png]
